# *Mycobacterium leprae* Induces Neutrophilic Degranulation and Low-Density Neutrophil Generation During Erythema Nodosum Leprosum

**DOI:** 10.3389/fmed.2021.711623

**Published:** 2021-10-08

**Authors:** Isabella Forasteiro Tavares, Jessica Brandão dos Santos, Fabiana dos Santos Pacheco, Mariana Gandini, Rafael M. Mariante, Thais Fernanda Rodrigues, Anna Maria Sales, Milton Ozório Moraes, Euzenir Nunes Sarno, Veronica Schmitz

**Affiliations:** ^1^Leprosy Laboratory, Oswaldo Cruz Institute, Oswaldo Cruz Foundation, Rio de Janeiro, Brazil; ^2^Laboratory of Cellular Microbiology, Oswaldo Cruz Institute, Oswaldo Cruz Foundation, Rio de Janeiro, Brazil; ^3^Laboratory of Structural Biology, Oswaldo Cruz Institute, Oswaldo Cruz Foundation, Rio de Janeiro, Brazil

**Keywords:** leprosy, *Mycobacterium leprae*, Erythema Nodosum Leprosum, neutrophilic degranulation, low-density neutrophils (LDNs), inflammation

## Abstract

Erythema Nodosum Leprosum (ENL) is a recurrent acute inflammatory complication of leprosy affecting up to 50% of all Borderline Lepromatous and Lepromatous Leprosy (BL/LL) patients. Although ENL is described as an immune reaction mediated by neutrophils, studies demonstrating the direct role of neutrophils in ENL are still rare. One subpopulation of low-density neutrophils (LDNs), present within the fraction of peripheral blood mononuclear cells (PBMC), has been associated with the pathogenesis and severity of diseases like sepsis, lupus, and tuberculosis. We herein analyzed LDNs and high-density neutrophils (HDNs) in terms of frequency, phenotype, and morphology. Serum levels of MMP-9 (a neutrophilic degranulation marker) were evaluated by ELISA; and LDNs were generated *in vitro* by stimulating healthy-donor, whole-blood cultures. PBMC layers of ENL patients presented segmented/hypersegmented cells that were morphologically compatible with neutrophils. Immunofluorescence analyses identified LDNs in ENL. Flow cytometry confirmed the elevated frequency of circulating LDNs (CD14^−^CD15^+^) in ENL patients compared to healthy donors and nonreactional Borderline Tuberculoid (BT) patients. Moreover, flow cytometry analyses revealed that ENL LDNs had a neutrophilic-activated phenotype. ENL patients under thalidomide treatment presented similar frequency of LDNs as observed before treatment but its activation status was lower. In addition, *Mycobacterium leprae* induced *in vitro* generation of LDNs in whole blood in a dose-dependent fashion; and TGF-β, an inhibitor of neutrophilic degranulation, prevented LDNs generation. MMP-9 serum levels of BL/LL patients with or without ENL correlated with LDNs frequency at the same time that ultrastructural observations of ENL LDNs showed suggestive signs of degranulation. Together, our data provide new insights into the knowledge and understanding of the pathogenesis of ENL while enriching the role of neutrophils in leprosy.

## Introduction

Leprosy, a chronic infectious disease caused by the intracellular, acid-fast bacillus *Mycobacterium leprae*, primarily affects the skin and peripheral nerves (1). The registered prevalence of leprosy (end of 2018) and new case detection worldwide in 2019 was recorded as 177,175 (WHO 2019). Clinical manifestations of leprosy are categorized in a wide-ranging spectrum according to the intensity of the individual immune response to infection (2).

Within the spectrum of the disease, Tuberculoid Leprosy (TT) is at the far end of the resistant pole and is characterized by few lesions, rare detectable bacilli, and an intense cellular immune response. At the opposite pole, however, Lepromatous Leprosy (LL) is diffuse, with intense bacillary multiplication and activation of humoral immunity in detriment of the cellular response. Borderline Tuberculoid (BT), Borderline Borderline (BB), and Borderline Lepromatous (BL), however, are unstable intermediate forms, classified as such according to their proximity to either end of the spectrum (2).

Erythema Nodosum Leprosum (ENL), an inflammatory complication of leprosy, affects 5–50% of all BL/LL patients and may occur at any time during multidrug therapy (MDT) (3, 4). ENL is clinically characterized by painful subcutaneous erythematous nodules and is most often accompanied by systemic symptoms such as malaise, fever, and neutrophilic leukocytosis (5). Classically, the presence of neutrophils in ENL lesions has diagnostically been considered an important factor. But more recent reports have highlighted the role of circulating neutrophils in ENL systemic inflammation, including their spontaneous ability to release TNF (6) and neutrophilic extracellular traps (7). Nonetheless, many gaps remain with respect to neutrophilic-*M. leprae* interaction.

One particular neutrophilic subpopulation presents in the peripheral blood mononuclear cell (PBMC) fraction in humans has been described and characterized. This subpopulation contains low-density neutrophils (LDNs), which are distinct from the classical and high-density neutrophils (HDNs) that are purified from the granulocytic fraction. High LDNs levels have been associated with the severity of lupus (8, 9); HIV infection (10); tuberculosis (11); sepsis (12); and cancer (13, 14). Although the origin of LDNs is still unclear, the main hypothesis is that they are activated neutrophils subject to degranulation, which is ultimately responsible for density loss (15, 16). In the present study, the aim was to identify and characterize both the presence of circulating LDNs during ENL and the ability of *M. leprae* to induce degranulation and generate LDNs.

## Methods

### Patients

This study was conducted at the Souza Araujo Outpatient Clinic, a reference center for leprosy diagnosis and treatment (Leprosy Laboratory, Oswaldo Cruz Foundation, Rio de Janeiro, RJ, Brazil). All leprosy patients were recruited with the approval of the Ethics Committee of the Oswaldo Cruz Foundation (CAAE 56113716.5.0000.5248). Leprosy diagnosis was based on the presence of hypopigmented, anesthetic skin patches; thickened nerves; and acid-fast bacilli in the skin smears based on Ridley and Jopling Scale criteria (2). Leprosy patients were treated with MDT, as recommended by the World Health Organization: rifampicin, dapsone, and clofazimine. Patients were diagnosed with ENL upon detection of an acute appearance of crops of tender cutaneous or subcutaneous lesions with or without any systemic symptoms and then treated with thalidomide (100 to 300 mg daily) in compliance with Brazilian Ministry of Health guidelines. Borderline Tuberculoid (BT), Borderline Lepromatous (BL), and Lepromatous Leprosy (LL) patients were also included in the study as control groups.

### ENL Severity

The present study measured the clinical severity of its ENL cases according to ENLIST ENL Severity Scale (17). The ENL Severity Scale contains a total of 10 items, namely: visual scale of pain; fever; the number, inflammation, and extent of ENL skin lesions; peripheral edema; bone pain; inflammation of joints and/or digits; lymphadenopathy; and nerve tenderness due to ENL.

### Blood Samples

Peripheral whole blood heparin samples were collected from all leprosy patients and endemic healthy controls enrolled in the study. Thalidomide-treated ENL patients had a scheduled second time point 7 days after initiating therapy (100–300 mg/day).

### Density Separation of LDNs and HDNs

Peripheral blood was diluted 1:1 with phosphate buffered saline (PBS, Gibco) and centrifuged on a cushion of Ficoll-Paque PLUS (GE Healthcare, USA) at a density of 1.076 g/mL in a centrifuge tube for 30 min at 400 × g at room temperature. PBMCs interfaces containing LDNs were collected and washed twice with PBS for 10 min at 300 × g, 4°C. HDNs were collected from the granulocytic-erythrocytic pellet by a brief hypotonic lysis of red blood cells by ACK solution (1.7 M NH_4_Cl; 0.1 M KHCO_3_; and 1 mM EDTA). HDNs were then washed twice with PBS and fixed in 4% paraformaldehyde. The cells were, respectively, suspended in PBS for immediate analysis by flow cytometry or other experiments. LDNs were identified as CD15^+^ CD14^−;^ and HDNs were identified as CD16^+^.

### Cytospin Slides

Samples containing (1 to 2 × 10^5^) LDNs or HDNs were resuspended in RPMI-1640 medium. The cells adhered to the slides by way of Shandon Cytospin 3 (Thermo Fisher, USA) at 150 × g for 6 min and then stained using the Fast Panoptic differential kit (Laborclin, Brazil). Cell morphology was analyzed by optical microscopy.

### Immunofluorescence

PBMCs adhered to slides by Cytospin, using 5 × 10^5^ cells per slide. The cells were fixed in 4% paraformaldehyde and washed in PBS. Permeabilization took place by using 0.5% saponin for 10 min in a humid chamber at 25°C. Unspecific binding sites were blocked with 5% Normal Goat Serum (NGS; Sigma-Aldrich, USA) in PBS for 30 min at 25°C. Cells were washed and labeled with Mouse anti-human PTX3 FITC antibody (Hycult, Netherlands) or the isotype diluted 1:50 with 5% Normal goat serum (NGS) in PBS for 1 h in a humid chamber in the dark. The nuclei were stained with 40–6-diamidino-2-phenylindole (DAPI; 1:10,000, Molecular Probes, D1306); and the slides were mounted with Permafluor (Thermo Fisher, USA) by placing a coverslip on the slide containing the cells. Lastly, the slides were sealed with Permount™ Mounting Medium and Fisher Chemical™/Fisher Scientific and subsequently analyzed under the AxiObserver Z1 Colibri microscope (Carl Zeiss, Germany). The images were processed *via* AxioVision software (Carl Zeiss, Germany).

### Transmission Electron Microscopy (TEM)

The fraction of PBMC containing 2 to 5 × 10^6^ cells was fixed in 2.5% glutaraldehyde (Sigma-Aldrich, USA) in 0.1 M sodium cacodylate buffer for 1 h at 25°C. The samples were then washed in the same buffer and post-fixed in 1% osmium tetroxide (Sigma-Aldrich, USA) containing 0.8% potassium ferrocyanide and 2.5 mM calcium chloride for 1 h at 4°C and later washed and dehydrated in acetone. Samples were embedded in PolyBed 812 resin (Polysciences, UK) and polymerized for 72 h at 60°C while ultrathin sections (60 nm) were obtained in an Ultracuts Ultramicrotome (Leica, Wien, Austria) and collected on copper grids. The sections were contrasted in 1% uranyl acetate and lead citrate for examination under the transmission electron microscope JEM-1011 (Jeol, Tokyo, Japan) at the Rudolf Barth Electron Microscopy Platform of the Oswaldo Cruz Institute/Fiocruz.

### Multiparametric Flow Cytometry

LDNs were analyzed in the fraction of PBMC. PBMCs or HDNs (5 × 10^5^ cells) were stained in the presence of Human FcR Blocking for 30 min at 4°C and protected from light with or without the following mixture of fluorescent-labeled, anti-human antibodies: CD14-FITC or PercP-Cy5.5; CD16-PE-Cy7; CD62L-FITC (eBioscience, USA); CD15-PE-Cy5 or Pacific Blue; and CD11b-PE (Biolegend, USA). Cells were washed with PBS/BSA 1% and fixed in 4% paraformaldehyde. The samples were stored protected from light in the refrigerator until the moment of acquisition. The BD FACSAria IIu cytometer (BD Biosciences, USA) was used to acquire the samples.

### Cytometric Analytical Strategy

Cytometric data were analyzed using FlowJo software (BD Systems, USA). As a first step, the singlet gate was performed with the forward scatter-height (FSC-h) vs. the respective FSC-area (FSC-a) to exclude doublets (cell aggregates). The gate was performed in the PBMC using the parameter side scatter-area (SSC-a) vs. FSC-a, thus eliminating cellular debris. LDNs were characterized within the PBMC gate as CD15^+^ CD14^−^ cells (15). Whereas, the CD14 marker is classically applied to identify monocytes, CD15 expression is traditionally used to identify peripheral neutrophils. CD11b, CD16, and CD62L expressions on LDNs were analyzed by median fluorescence intensity (MFI). Fluorescence Minus One (FMO) was adopted as a control sample without labeling the receptors of interest. The MFI value of the control FMO sample was subtracted from the MFI of the recently-analyzed labeled sample to add to the data in the graphs.

### ELISA

Concentrations of Matrix-Metalloproteinase-9 (MMP-9) were calculated in serum samples *via* a commercial enzyme-linked immunosorbent assay kit (ELISA), according to the manufacturer's protocol (DY911—R&D Systems, USA). This assay detects total MMP-9 in both its active and *pro forma* form. Values represent the mean of duplicate determinations in each sample and the absorbance was compared with the standard curve to calculate concentration rates. Results were expressed in pg/mL after processing the data using SoftMax Pro software, version 4.8 (Molecular Devices, USA).

### Generation of LDNs *In vitro*

Two milliliters of whole blood from healthy donors were incubated in the absence or presence of sonicated dead *M. leprae* (BEI Resources, USA) at concentrations of 2.5, 5, and 10 μg/mL, y-irradiated *M. leprae* (BEI Resources, USA) at 10 μg/mL and Lipopolysaccharide (LPS) (Sigma-Aldrich, USA) at 100 ng/mL for 24 h at 37°C, 5% CO_2_. In some control cultures, 10 ng/mL of the human recombinant TGFβ1 (Peprotech, Germany) were incubated during stimuli. The whole blood was then fractionated by Ficoll-Paque density gradient centrifugation, as previously mentioned. LDNs within the PBMC fraction were analyzed by flow cytometry by using the same gated strategy already described.

### Statistical Analyses

The analyses were performed using GraphPad PRISM software, version 6 (GraphPad Software, USA). Data are presented as medians (interquartile range [IQR]). The D'Agostino-Pearson normality test was applied to observe data distribution. To compare the results of the two groups, the unpaired Mann–Whitney test and the Wilcoxon test for paired samples of non-parametric distribution were utilized. To contrast more than two groups of non-parametric samples, the Kruskal–Wallis test followed by Dunn's multiple comparison test were carried out. Spearman or Pearson correlation coefficients were used when appropriate, depending on data distribution. The differences were considered significant when *p* < 0.05.

## Results

### Patient Clinical Background

Demographic and clinical information of the patients and controls enrolled in the present study is included in Table 1. The study population consisted of 98 leprosy patients (24 females and 74 males; Median age [IQR]: 46 [34–53] years). Patients were divided into two groups. The first contained 36 BL/LL patients with ENL (8 females and 28 males; Median age [IQR]: 40 [33–52] years). The second was the control group of leprosy patients with no history of ENL and/or other types of leprosy reactions, comprising 31 BL/LL (8 females and 23 males; Median age [IQR]: 45 [32–52] years) and 13 BT patients (5 females and 8 males; Median age [IQR]: 51 [39–61] years). Leprosy patients without any reactions were included before starting MDT. In addition, as previously referred to, 18 ENL patients were analyzed 7 days after starting thalidomide treatment (ENL THAL). Six endemic healthy controls (4 females and 2 males; Median age [IQR]: 37 [28–45] years) were also examined.

**Table 1:** Demographic and clinical patient data.

**Characteristics**	**BT**	**BL/LL^a^**	**ENL^b^**	**ENL THAL^c^**
Individuals, No.	13	31	36	18
Sex				
Female	5	8	8	3
Male	8	23	28	15
Median age, years (IQR)	51	45	40	46
	39–61	32–52	33–52	37–53
BI (Median)	0	4.87	3.75	–

a *BL and LL patients without reaction*.

b *BL and LL patients with ENL*.

c *ENL THAL, ENL patients 7 days after starting thalidomide treatment*.

*IQR, Interquartile range; BI, Bacillary Index; min, minimum; max, maximum*.

### Identification of LDNs in the Peripheral Blood of ENL Patients

**Figure 1:**
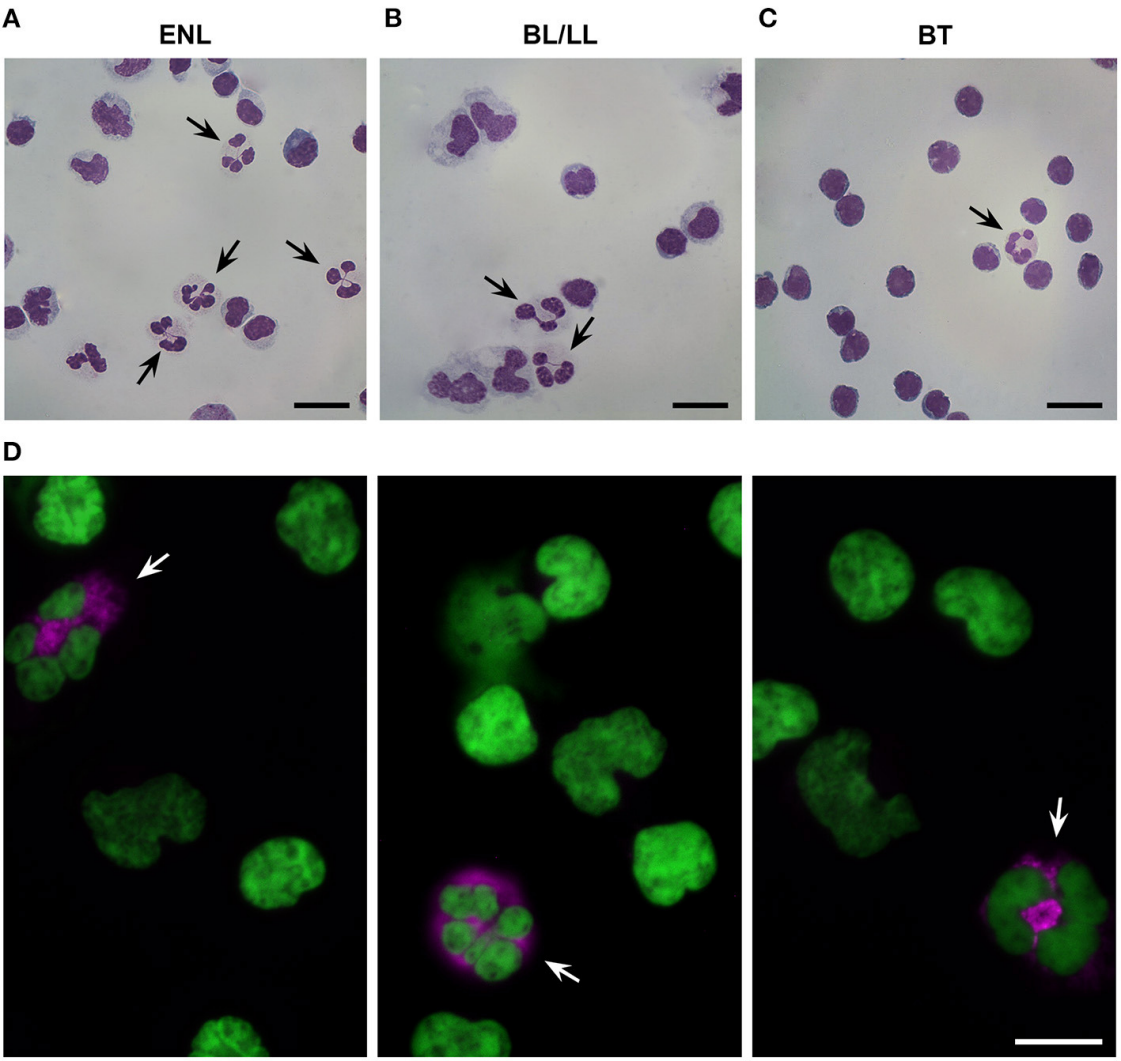
Presence of low-density neutrophils in the peripheral blood of ENL patients. **(A)** Representative image of low-density neutrophils (LDNs) with a segmented nucleus within the peripheral blood mononuclear cell (PBMCs) layer of ENL patients (*n* = 4). **(B)** BL/LL patients (*n* = 3) and **(C)** BT patients (*n* = 2). **(D)** Representative image of immunofluorescence in the PBMC layer of an ENL patient (*n* = 3). LDNs were considered pentraxin-3-positive (PTX3+) cells. PTX3 (pink) and nucleus (DAPI, green). Arrows indicate LDNs. Scale bar = 10 μm.

Morphological analysis revealed segmented neutrophilic-like cells in the PBMC fractions of ENL patients (Figure 1A). Immunofluorescence analysis further confirmed the presence of LDNs in ENL PBMCs, as shown by cells with a segmented nucleus and positive staining for PTX3, a protein stored selectively in specific neutrophilic granules (18) (Figure 1D). Qualitative analyzes revealed few LDNs in PBMC fractions from both BT and BL/LL patients (Figures 1B,C, respectively).

**Figure 2:**
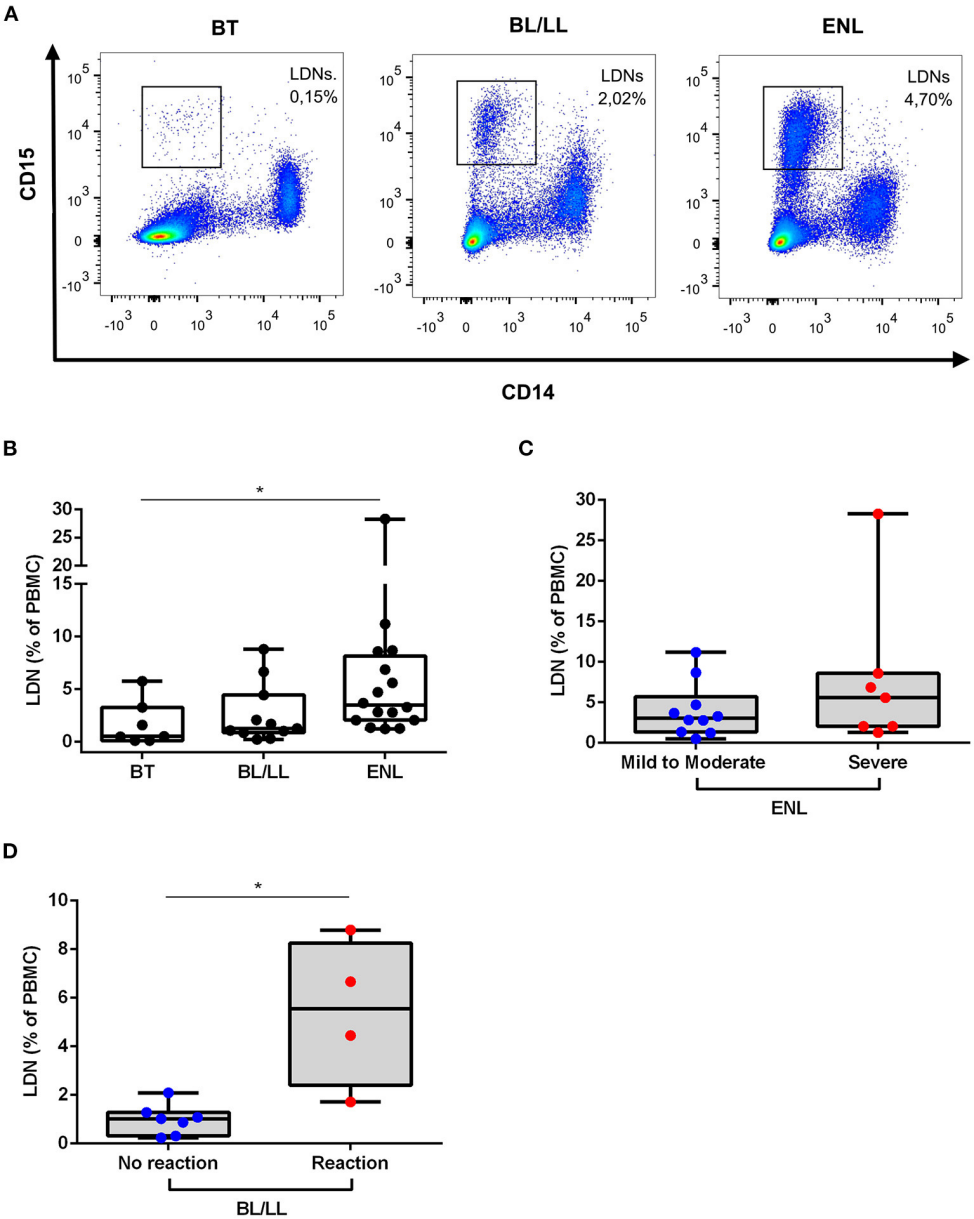
Low-density neutrophils are elevated in ENL patients. **(A)** Representative flow cytometric analysis of low-density neutrophils (LDNs) in peripheral blood mononuclear cells (PBMCs) of BT, BL/LL, and ENL patients. LDNs were gated within the PBMCs as CD14^−^CD15^+^ cells. **(B)** Frequency of LDNs in the PBMC layer of BT (*n* = 7), BL/LL (*n* = 11), and ENL patients (*n* = 16). **(C)** Frequency of LDNs in the PBMC layer of ENL patients classified with mild-to-moderate (*n* = 9) and severe ENL (*n* = 7) according to the ENList scale. **(D)** Frequency of LDNs on the PBMC layer of BL/LL patients who did not develop reaction (*n* = 7) and BL/LL patients who developed any type of leprosy reaction up to one year after the analyses (*n* = 4). Borderline tuberculoid (BT), borderline lepromatous/lepromatous leprosy (BL/LL), and erythema nodosum leprosum patients (ENL). Box plots show median, interquartile range, and sample minimum and maximum. Dots represent individual patients. Statistic: **(B)** Kruskal–Wallis with Dunn's post-test and **(C,D)** Mann–Whitney. *p < 0.05.

To confirm and quantify the presence of circulating LDNs in the leprosy patients, multiparametric flow cytometry was used based on the expressions of CD14 and CD15. The overall gate strategy is presented in Supplementary Figure 1A. As noted in previous reports, LDNs were identified as CD14^−^CD15^+^ cells (11, 15) while, not surprisingly, endemic controls presented marginal frequency of LDNs (medians [IQR]: 0.28 [0.12–0.43]%) (Supplementary Figures 1A,B). All leprosy patients presented enriched levels of LDNs (Figures 2A,B). PBMCs of ENL leprosy patients contained elevated percentages of LDNs compared with the PBMCs of the BT (medians [IQR]: 3.49 [2.06–8.15]% vs. 0.53 [0.13–3.27]%, *p* = 0.03; respectively) and BL/LL controls (medians [IQR]: 3.49 [2.06–8.15]% vs. 1.28 [0.87–4.45]%; respectively). The minimum and maximum frequencies of ENL LDNs in the present study were, respectively, 1.24 and 28.3%. No significant difference was found in LDNs frequency between BT and BL/LL patients (medians [IQR]: 0.53 [0.13–3.27]% vs. 1.28 [0.87–4.45]%; respectively).

Since ENL patients presented a huge variation in levels of LDNs, the relation between LDNs frequency in the blood and the severity of ENL based on the ENLIST scale (17) was investigated. Of all ENL patients analyzed for LDNs frequency, 9 were classified as having mild-to-moderate ENL and 7, with severe ENL. In Figure 2C, no significant difference can be seen among the frequency of LDN in severe ENL patients when compared to mild-to-moderate ENL ones (medians [IQR]: 5.60 [2.06–8.58]% vs. 3.29 [2.08–6.70]%, respectively). The frequency of circulating LDNs in BL/LL patients was examined by comparing those who developed any type of leprosy reaction with those who did not. After 30 months of follow up (Figure 2D), it was found that the BL/LL patients who developed reaction presented significantly higher LDNs levels at the moment of leprosy diagnosis than the BL/LL patients who did not develop any reaction (medians [IQR]: 1.020 [0.31–1.28]% vs. 5.56 [2.40–8.26]%, respectively, *p* = 0.01).

### Characteristics of LDNs in ENL Patients

**Figure 3:**
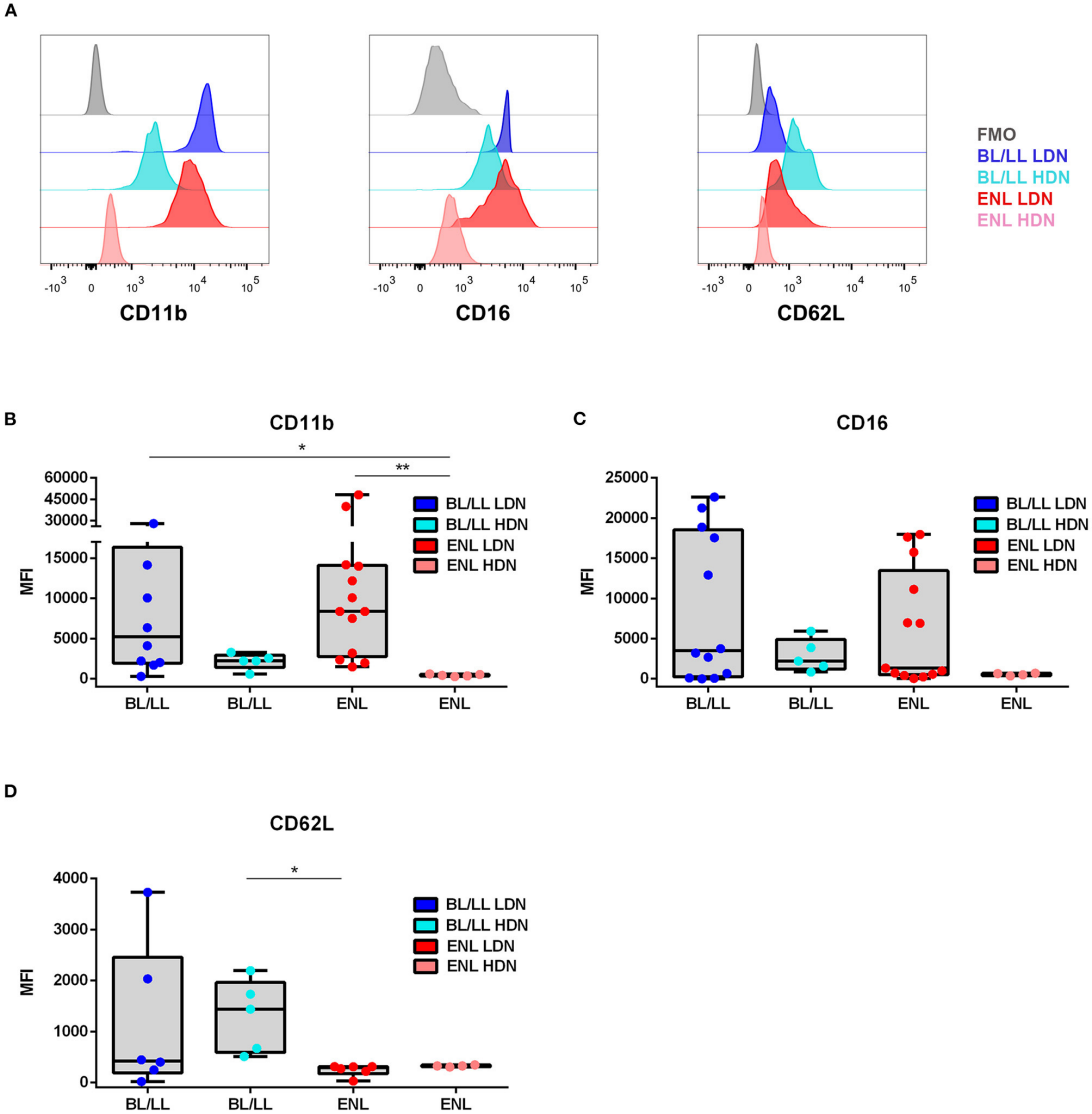
Phenotypic profile of LDNs and HDNs of BL/LL patients with or without ENL. **(A)** Representative FACS analyses of CD11b, CD16, and CD62L surface expressions of leprosy patients. The mean fluorescence intensity (MFI) was calculated by subtracting the MFI from the FMO control sample of each marker in LDNs and HDNs cells. **(B)** CD11b expression in BL/LL (LDN *n* = 10; HDN *n* = 5), and ENL (LDN *n* = 13; HDN *n* = 5) patients. **(C)** CD16 expression in BL/LL (LDN *n* = 12; HDN *n* = 5); and ENL (LDN *n* = 13; HDN *n* = 4) patients. **(D)** CD62L expression in BL/LL (LDN *n* = 6; HDN *n* = 5) and ENL (LDN *n* = 6; HDN *n* = 4) patients. Borderline Lepromatous/Lepromatous leprosy (BL/LL) and erythema nodosum leprosum patients (ENL). Box plots show median, interquartile range, sample minimum, and maximum. Dots represent individual patients. Statistic: Kruskal–Wallis with Dunn's post-test. *p < 0.05, **p < 0.01.

**Figure 4:**
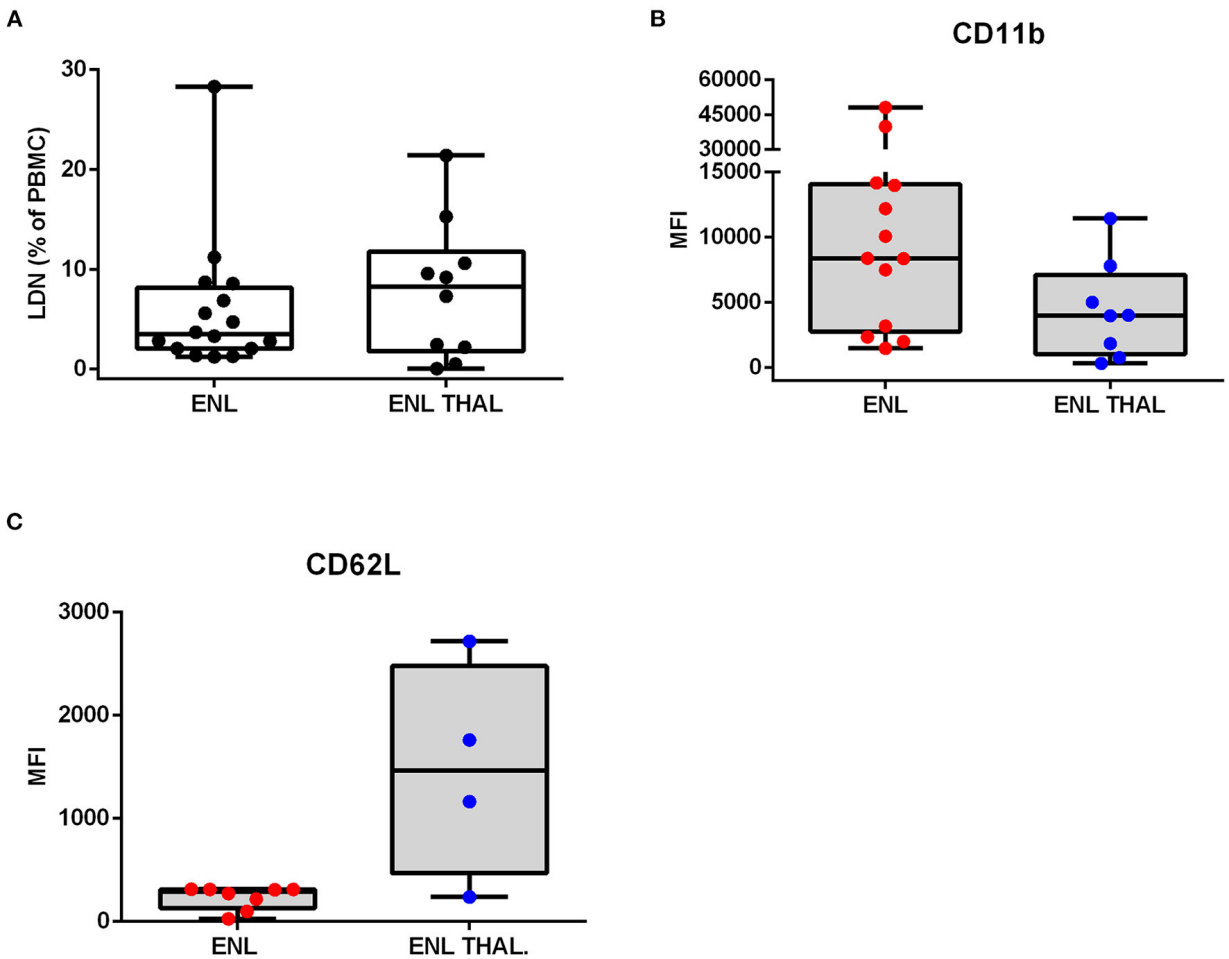
Frequency and phenotypic analyses of low-density neutrophils in ENL patients undergoing thalidomide treatment. ENL patients were analyzed at diagnosis (ENL) and seven days after starting thalidomide treatment (ENL THAL). **(A)** Frequency of LDNs in the PBMC layer of ENL (*n* = 16) and ENL THAL (*n* = 10) patients. MFI was calculated by subtracting the MFI from the FMO control sample for each LDNs marker. **(B)** CD11b expression on ENL (*n* = 13) and ENL THAL (*n* = 8) patients. **(C)** CD62L expression on ENL (*n* = 8) and ENL THAL (*n* = 4) patients. Box plots show median; interquartile range; and sample minimum and maximum. Dots represent individual patients. Statistic: **(A–C)** Mann–Whitney.

Phenotypic analysis of neutrophilic surface markers such as CD11b, CD16, and CD62L revealed significant differences between LDN and HDN subpopulations (13, 14, 16). In this context, it was evaluated whether the LDNs and HDNs of ENL patients were phenotypically different from the ones pertaining to BL/LL patients by comparing their MFI (Figure 3A). The Fluorescence Minus One Control (FMO) was adopted as the overall gate strategy (Figure 3A). The mean expression of the integrin CD11b on the LDNs of ENL patients was higher than on HDNs of ENL patients (medians [IQR]: 8385 [2,777–14,088] vs. 447.9 [331.9–571.9], respectively; *p* = 0.002; Figure 3B). LDNs of BL/LL patients also had increased MFI as opposed to the HDNs of ENL patients (medians [IQR]: 5,242 [1,962–16,372] vs. 447.9 [331.9–571.9], respectively; *p* = 0.03; Figure 3B). It was not significant the difference of the mean expression of CD11b on LDNs of ENL patients than on LDNs of BL/LL ones (medians [IQR]: 8,385 [2,777–14,088] vs. 5,242 [1,962–16,372], respectively; Figure 3B). The CD16 expression remained the same in all of the analyzed groups (Figure 3C). Regarding CD62L expression, a reduction was observed in LDNs of ENL patients compared to HDNs of BL/LL patients (medians [IQR]: 289 [171–310.8] vs. 1,440 [589.4–1,963], respectively; *p* = 0.01) (Figure 3D). No difference was found between the frequency of LDNs in the PBMCs of ENL patients who had been taking thalidomide for 7 days (ENL THAL) or ENL patients at the moment of diagnosis (medians [IQR]: 8.25 [1.78–11.78]% vs. 3.49 [2.06–8.153]%, respectively; Figure 4A). Our data also did not show significant differences among the expression of the activation markers analyzed comparing newly-diagnosed ENL and ENL THAL patients (CD11b medians [IQR]: 8,385 [2,777–14,088] vs. 4,222 [1,052–7,120]%; CD62L medians [IQR]: 289.0 [129.0–310.8] vs. 1,463 [469.3–2,479]) (Figures 4B,C).

### *M. leprae* Induced the Generation of LDNs in a Dose-Dependent Manner

**Figure 5:**
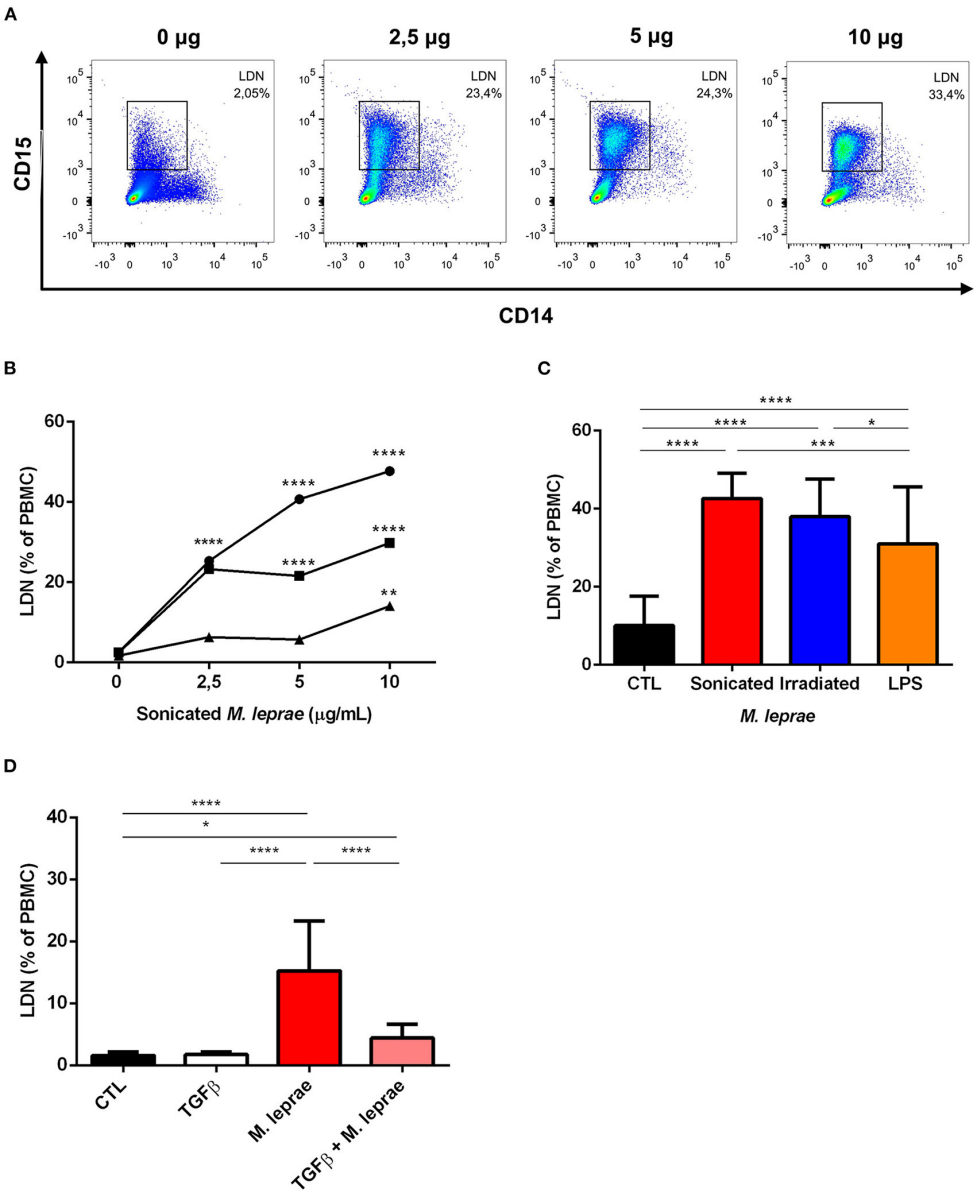
*M. leprae* induces low-density neutrophilic generation *in vitro*. **(A)** Representative FACS analysis of a whole blood sample of a health donor. The sample was stimulated with 0, 2.5, 5, and 10 μg/mL of sonicated *M. leprae* (*n* = 3). After 24 h of incubation, the culture cells were submitted to density centrifugation and the PBMC layer was analyzed by flow cytometry to identify CD14^−^CD15^+^ LDN. **(B)** LDNs frequency in healthy-donor whole blood cultures stimulated or not with sonicated *M. leprae* at different concentrations (2.5, 5, and 10 μg/mL) for 24 h incubation (*n* = 3). **(C)** Frequency of LDNs in peripheral blood of healthy individuals stimulated with 10 μg/mL of sonicated or irradiated *M. leprae* and 100 ng/mL of LPS (*n* = 3). **(D)** Frequency of LDNs in the peripheral blood of healthy individuals stimulated with TGFβ1 at 10 ng/mL alone or in combination with 10 μg/mL of sonicated *M. leprae* (*n* = 3). All experiments were carried out in triplicate. Statistics: two-way ANOVA with Sidak's post-test. *p < 0.05, **p < 0.01, ***p < 0.001, and ****p < 0.0001.

The previously-described results demonstrated that ENL patients had significantly elevated levels of LDNs in their peripheral blood, suggesting that *M. leprae* either induces the generation of LDNs or promotes the conversion of HDNs into LDNs. To confirm the correlation of *M. leprae* stimulation with the elevated LDNs levels, peripheral blood of healthy volunteers was collected and then challenged *in vitro* with sonicated *M. leprae* for 24 h, subjected to density centrifugation, and analyzed for LDNs levels by flow cytometry, as demonstrated above. It was initially observed that the HDN-to-LDN transition occurred spontaneously in non-stimulated cultures after a 24-hr incubation period (Figures 5A,B). Our results showed that LDNs levels were significantly elevated following the *M. leprae* challenge in a dose-dependent manner (Figures 5A,B). Furthermore, the potential of irradiated *M. leprae* and LPS, a positive control favoring LDNs generation in whole blood, was also analyzed (Figure 5C), demonstrating that irradiated *M. leprae* and LPS were also able to induce LDNs *in vitro* (Figure 5C).

### TGF-b Regulated the Transition From HDN to LDN

As seen above, some studies suggest that degranulation is the key mechanism involved in the etiology of LDNs (10, 19). Conversely, TGF-β has been pointed to as an inhibitor of neutrophilic degranulation (20). As such, the effect of TGF-β on the generation of LDNs mediated by sonicated *M. leprae* was evaluated. When compared to an unstimulated control, TGF-β alone showed no results (Figure 5D). Nevertheless, the presence of TGF-β together with sonicated *M. leprae* was able to reduce the generation of LDNs by *M. leprae* (Figure 5D).

### Involvement of Degranulation in the Generation of LDNs During ENL

**Figure 6:**
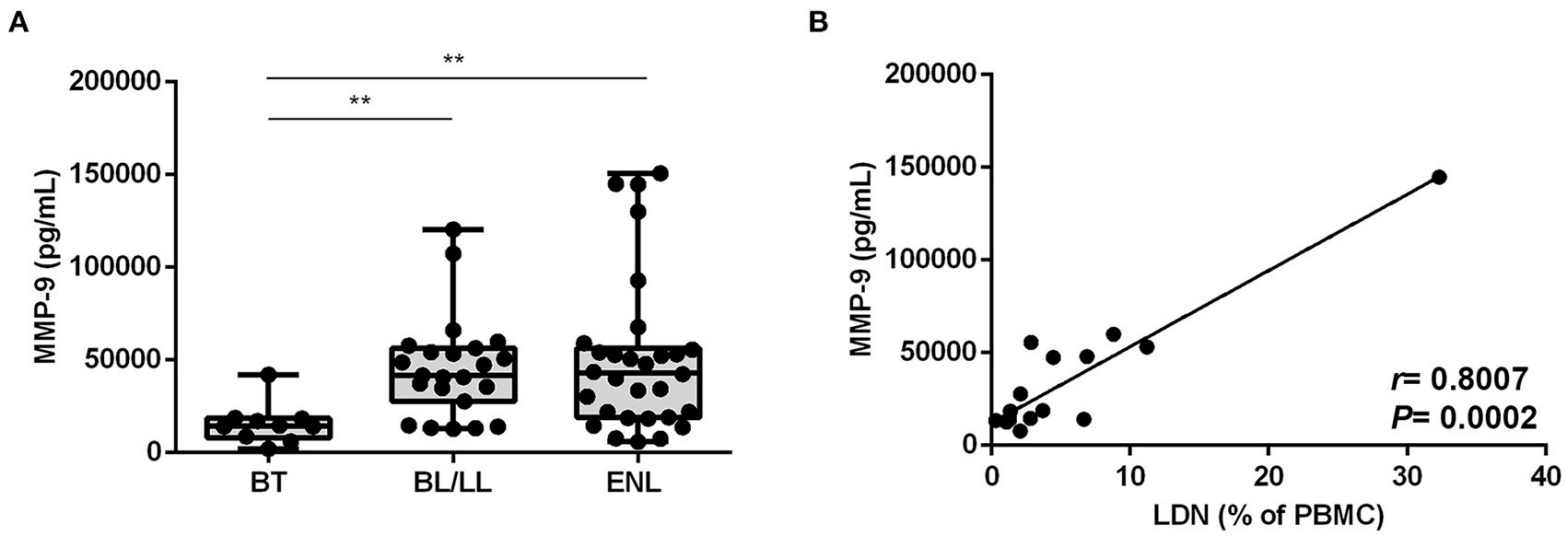
MMP-9 degranulation correlates with the frequency of LDNs in BL/LL and ENL patients. **(A)** MMP-9 concentration was evaluated by enzyme-linked immunosorbent assay (ELISA) in the sera of 10 BT, 24 BL/LL, and 31 ENL patients. Box plots show median; interquartile range; and sample minimum and maximum. Dots represent individual patients. **(B)** Correlation between MMP-9 serum levels and frequency of circulating LDNs on BL/LL patients with or without ENL (*n* = 17). Statistic: **(A)** Kruskal–Wallis with Dunn's post-test and **(B)** Spearman's rank correlation coefficient (R). **p <0.01.

Next, to observe evidence of degranulation *in vivo*, the serum levels of MMP-9, a protein present in tertiary neutrophilic granules, were investigated. MMP-9 serum levels of BT patients were significantly lower than among BL/LL and ENL patients (BT median [IQR]: 14,329 [8,087–18,539], BL/LL median [IQR]: 41,720 [27,753–56,584], ENL median [IQR]: 42,987 [19,045–56,396] pg/mL) (Figure 6A). In fact, there was no difference between the BL/LL patients with or without ENL (Figure 6A) although a positive correlation (*r* = 0.8007, *P* = 0.0002) between LDNs frequency and serum MMP-9 levels in leprosy patients was found (Figure 6B).

**Figure 7:**
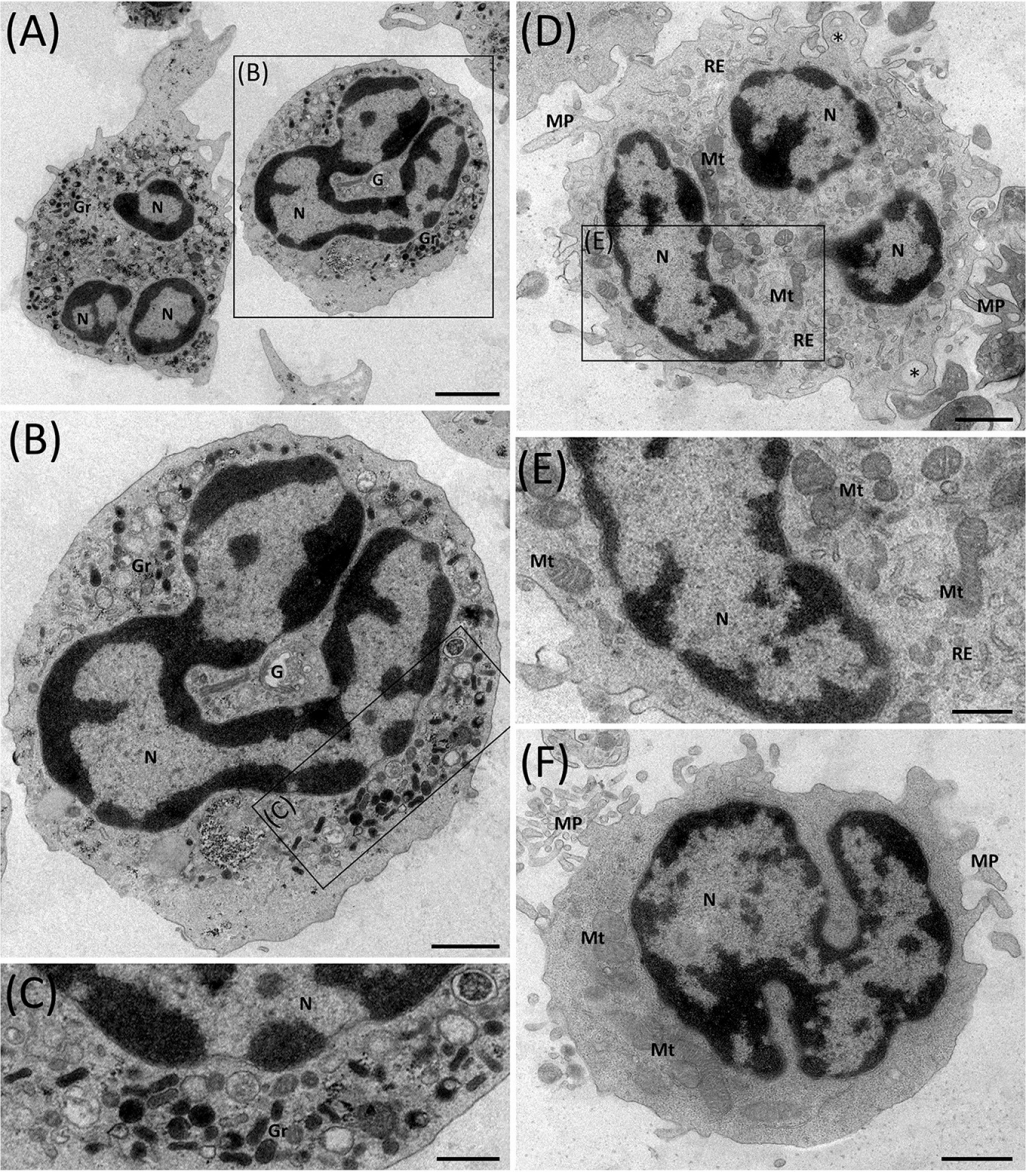
Ultrastructure of ENL HDNs and LDNs as seen *via* transmission electron microscopy. **(A–C)** HDNs contain typical lobulated nuclei (N) with a large amount of highly condensed heterochromatin (electron-dense region) at the periphery and various types of cytoplasmic granules (Gr), variable in size, shape, and electron-density. Image representative of three (*n* = 3) ENL patients. **(D–F)** LDNs possess lobulated nuclei, very few cytoplasmic granules, several mitochondrial (Mt), and endoplasmic reticulum (ER) profiles, membrane projections (MP), and empty vesicles (asterisks). Images representative of six (*n* = 6) ENL patients. G, Golgi complex. Bar = 2 μm **(A)**, 1 μm **(B,D,F)** and 500 nm **(C,E)**.

To investigate whether the degranulation process mediates the change in neutrophilic density, transmission electron microscopy (TEM) was used, focusing on ENL patients HDNs and LDNs. The presence of segmented nuclei, with well-defined regions of heterochromatin (electron-dense) and euchromatin (electron-lucent; Figure 7), were displayed in the HDNs and LDNs. Contrariwise, no vacuolization in the cell cytoplasm nor signs of apoptosis in any of the subpopulations were detected. After a qualitative analysis, however, the granules were found to be much less evident in the cytoplasm of LDNs (Figures 7D–F) than in HDNs (Figures 7A–C). In addition, while hardly ever seen in HDNs, a large number of mitochondrial and endoplasmic reticulum profiles in conjunction with membrane projections was evident in the LDNs (Figures 7A–F).

## Discussion

To date, neutrophils have been little studied within the context of leprosy disease. This might be so because neutrophils, unlike macrophages and Schwann cells, are not the prime targets of *M. leprae per se* (21). The results reported in the present study, however, aim to provide further insight into several key aspects of LDNs in leprosy. An initial consideration is that the LDNs levels of ENL patients were found to be distinctly higher than those among patients without ENL. Moreover, the LDNs of ENL patients presented elevated levels of CD11b, decreased levels of CD62L, and similar levels of CD16 in comparison to HDNs, all suggestive of a more robust activation state (22). Whereas the frequency of LDNs remained unaltered in the ENL patients undergoing a week-long thalidomide treatment, the surface expressions of CD11b and CD62L, decreased and increased, respectively. Thus, LDNs in ENL appears to display an activated or primed neutrophilic phenotype, similar to that of the LDNs previously described in patients with tuberculosis, autoimmune diseases, and HIV infection (11, 23, 24).

To our knowledge, the present study showed, for the first time in the literature, the ability of *M. leprae* to induce LDNs generation in the whole blood of healthy individuals. Our study also proposes that the presence of *M. leprae* is an important factor in triggering the onset of *in vivo* LDNs. Indeed, it was clearly demonstrated that certain factors present in MB patient serum induced the presence of granulocytes in the PBMC layer of healthy individuals (25). It was also found that the generation of LDNs following *M. leprae* stimulation is dose-dependent. Due to the difficulty in obtaining laboratory-grown *M. leprae*, the present study was unable to ascertain if *M. leprae* infection can induce the generation of LDNs. It has been previously shown that the LDNs levels induced by live *M. tuberculosis*, e.g., are significantly lower in comparison to those induced by heat-killed mycobacteria (11).

Our results have presented both *in vivo* and *in vitro* evidence of the ability of *M. leprae* to induce neutrophilic degranulation. Even so, whether this ability involves direct or indirect action on the part of the pathogen remains elusive, especially since *M. leprae* has been found capable of inducing cytokine production in neutrophils *in vitro* (6). The higher serum levels of MMP9 and their correlation with the frequency of LDNs corroborated the *in vitro* data showing that the addition of TGF-β, an inhibitor of degranulation, to whole blood cultures nullifies LDNs induction by dead *M. leprae*. Furthermore, LDNs induction by *M. leprae via* degranulation is reinforced by the evidence that pentraxin 3 (PTX3), another neutrophilic protein granule, is released systemically as well as at the site of ENL lesions (26) and the neutrophilic degranulation signature observed in ENL lesions (27). Increased MMP-2, MMP-9 mRNA, and protein MMP-9 levels were detected together with enhanced TNF-α and IFN-γ expression levels in the lesions of both tuberculoid patients and in those undergoing leprosy reactions like ENL (28). It is worth highlighting that TNF is known to induce MMP-9 degranulation of neutrophilic tertiary granules (29). These findings, along with the capacity of circulating ENL neutrophils to spontaneously release TNF (6), provide valuable clues regarding the possible mechanisms involved in degranulation in leprosy.

Ultrastructural observations of neutrophils in ENL also infer that degranulation is the source of LDNs in leprosy. As seen by TEM, LDNs contain almost no cytoplasmic granules. In addition, empty vesicles, suggestive of degranulation (30), can be seen in the cytoplasm of LDNs, but not in HDNs, implying that the former may be derivatives of degranulated neutrophils (31). Surprisingly, LDNs presented several profiles of mitochondrial and endoplasmic reticulum (ER) as well as many projections of the cell-surface membrane, barely found at all HDNs. Whether, the biogenesis of mitochondria reflects the activation status of neutrophils, as is the case of other cell types such as CD4^+^ T lymphocytes (32), deserves further investigation. But it is the belief of the present authors that mitochondrial biogenesis may represent an important step in the degranulation process since reactive oxygen species derived from mitochondria directly affect neutrophilic activation and function, which includes degranulation (33). Notably, ER stress has been shown to increase neutrophilic degranulation (34, 35), suggesting that changes in ER biogenesis may compromise the performance of neutrophilic functions, including degranulation itself.

Previous studies have associated LDNs with severity in tuberculosis and in sepsis (11, 12). It is herein demonstrated that the ENL patients classified as severe had a higher frequency of circulating LDNs when compared to those with mild-to-moderate symptoms. These data require further verification with a much larger group of cohorts to validate or not any correlation between severe ENL and LDNs. Interestingly, heretofore non-reactional BL/LL patients who developed reactions soon after the analyses had increased LDNs levels. This result implies that high levels of LDNs may be considered potential predictive markers of ENL reaction. Likewise, the presence of LDNs in the circulation of ENL patients was more robust.

The systemic inflammation associated with ENL is one of the most important aspects related to leprosy morbidities. In addition, despite some advances in the understanding of leprosy immunopathology, the mechanisms that might trigger ENL have not been sufficiently explored. The exclusive presence of neutrophils in ENL lesions has always intrigued pathologists and scientists as it is unknown whether neutrophils are a cause or, rather, a consequence of the inflammatory reaction. Again, in the present study, a large number of clinical specimens taken from ENL patients during reaction confirmed the participation of LDNs in the disease. Higher CD11b and lower CD62L surface expressions on LDNs correlate with the activation status of LDNs. Indeed, the detection of higher LDNs frequency in severe ENL all but reinforces the role played by LDNs in ENL pathogenesis. As a final point, MMP9 serum analyses in conjunction with morphological observations lead to degranulation as the source of LDNs in leprosy. Overall, these results pave the way for the development of new therapeutic interventions during ENL.

## Data Availability Statement

The raw data supporting the conclusions of this article will be made available by the authors, without undue reservation.

## Ethics Statement

The studies involving human participants were reviewed and approved by Ethics Committee of the Oswaldo Cruz Foundation (CEP IOC/Fiocruz). The patients/participants provided their written informed consent to participate in this study.

## Author Contributions

VS: designed research. VS, MM, and ES: obtained funding. AS and VS: enrolled patients, performed, and registered clinical diagnosis. IT, VS, JdS, FP, TR, and RM: performed the following experiments. IT, VS, RM, and MG: analyzed the data. IT and VS: wrote the manuscript. IT, VS, JdS, FP, MG, RM, AS, MM, and ES: critically revised the manuscript and approved its final version. All authors contributed to the article and approved the submitted version.

## Funding

This study was supported by grants from the Oswaldo Cruz Foundation (FIOCRUZ). ES and MM are research fellows of the Brazilian Research Council (CNPq). The funders had no role in study design, data collection and analysis, decision to publish, or preparation of the manuscript. IT is the recipient of a fellowship from CNPq. JdS and TR are the recipient of a fellowship from Coordenação de Aperfeiçoamento de Pessoal de Nível Superior (CAPES).

## Conflict of Interest

The authors declare that the research was conducted in the absence of any commercial or financial relationships that could be construed as a potential conflict of interest.

## Publisher's Note

All claims expressed in this article are solely those of the authors and do not necessarily represent those of their affiliated organizations, or those of the publisher, the editors and the reviewers. Any product that may be evaluated in this article, or claim that may be made by its manufacturer, is not guaranteed or endorsed by the publisher.
