# Supplementary material for: Mycobacterium leprae Induces Neutrophilic Degranulation and Low-Density Neutrophil Generation During Erythema Nodosum Leprosum
Source: Front Med (Lausanne). 2021 Oct 8;8:711623. doi: 10.3389/fmed.2021.711623 (PMC8531262; doi:10.3389/fmed.2021.711623)
Supplement: Supplementary file 1 [file Data_Sheet_1.PDF]

SUPPLEMENTARY MATERIAL

(A)

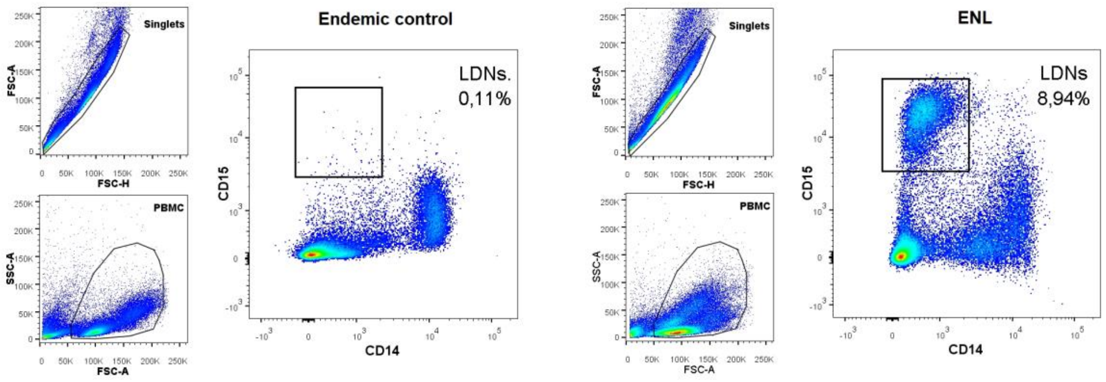

(B)

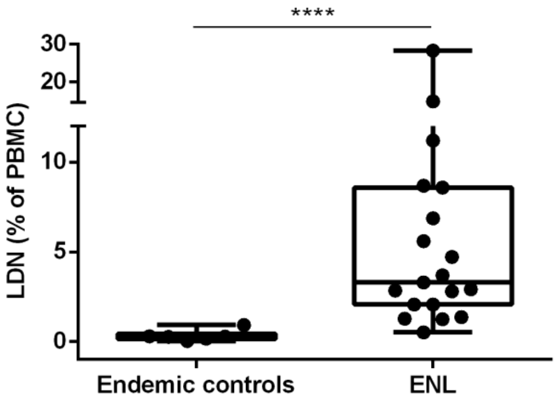

Figure S1: Flow cytometric analysis strategy on endemic controls and ENL patients. (A) Representative flow cytometric analysis of low-density neutrophils (LDNs) in peripheral blood mononuclear cells (PBMCs) of endemic healthy controls and ENL patients. Singlets were gated using the FSC-A x FSC-H parameters. Within the singlets, PBMCs were gated according to SSC-A x FSC-A. LDNs gates were made within the PBMCs as $CD14^{-}CD15^{+}$ cells. (B) Frequency of LDNs in the PBMC layer of endemic healthy controls (n=6) and ENL patients (n=16). Erythema nodosum leprosum patients (ENL). Box plots show median; interquartile range; and sample minimum and maximum. Dots represent individual patients. Statistic: Mann-Whitney. ****p < 0.0001.
